# Psychological Fragments

**Published:** 1849-10-01

**Authors:** 


### Psychological Fragments.

PROGRESSIVE GENERAL PALSY.—In the *Annales Medico-Psychologiques*, M. Lunier has published a paper on progressive general palsy, which he seeks to show may affect the sound in mind, as well as the insane. He says its nature was misunderstood until towards the close of the last century; it was described by Haslam, Esquirol, and Georget, as a complication or rather a termination of insanity. Progressive general palsy was for a long while, and is even now, regarded by many practitioners as a disease proper to the insane. Nevertheless, one of the earliest works published on this subject,—the thesis of M. Delaye, which appeared in 1824, contains a case of progressive general palsy, which occurred in an individual whose intellect was perfectly sound. The author of this excellent dissertation, however, considers the case in question to be exceptional, and the greater number of practitioners who have since written on the subject, speak of it as the only case that has come to their knowledge. The name of general palsy of the insane, which is used to designate the disease, serves to propagate and maintain the error. A few more cases, he observes, have since been published, illustrative of the view he takes, and he adds that, if we read attentively the facts brought forward by J. Bayle, Delaye, Calmeil, Foville, Deveau, Lelut, Parchappe, Wachter, Requin, &c., and the excellent description many of these authors have given of general palsy, it will be easy to recognise that this morbid state, which they have described as a symptom or termination of insanity, existed in many of their cases before there was any lesion of the intellectual faculties, and that the lesion occurred most frequently consecutively only, and as a complication of the paralysis.

M. Lunier seeks to establish that progressive general palsy may be met with in the general hospitals, although not so frequently as in lunatic asyla; that the palsy, in the one case, in no respect differs from that in the other; that the lesions of the intellectual faculties met with in such cases do not deserve generally the name of insanity, but consist simply in a diminution, or an abolition, or a more or less complete palsy of these faculties, comparable to the palsy of motion and sensation, and finally that progressive general palsy constitutes a special and clearly defined disease, which ought to be completely separated from insanity, just as are epilepsy and hysteria.

His essay is divided into three parts; the first treats of those cases in which the progressive general palsy has not been preceded nor accompanied by insanity or dementia; the second describes those cases where the palsy, without having been preceded by lesions of the intellectual faculties, has been afterwards complicated with dementia; and in the third will be brought forward those cases in which the general palsy has been or seems to have been preceded by mania or monomania, transformed or not afterwards into dementia.

Progressive general palsy rarely reaches its second stage, or even the termination of its first, without the occurrence of symptoms of dementia. It is only at the commencement of the disease that there is not found any lesion of the intellectual faculties. These cases are more frequently met with in private practice. The absence of all lesion of the intellect at the commencement of general palsy renders the diagnosis of the disease difficult. The symptoms of dementia are generally those first recognised by the relatives or friends of the patient, and when a medical man is called in, he is astonished to find the palsy already much advanced, and most frequently completely incurable.

The first part contains the details of six cases; in the first, that of a man named Lenoir, fifty-six years of age, the disease was hereditary, and the patient himself was subject to attacks of cerebral congestion, for which he had been in the habit of losing blood—a practice he had latterly neglected; besides which, he had had a rather free hemorrhoidal flux, which had ceased of late. Lenoir presented almost all the symptoms of the first stage of progressive general palsy; weakness of the special sensations and of the general sensibility, hesitation in walking, diminution of the strength of the upper limbs, a slight embarrassment in speaking, loss of the generative power, fleeting attacks of giddiness; there was not, however, any affection of the intellectual faculties. The treatment consisted in the abstraction of blood, the administration of purgatives, low diet, and rest. Some of the symptoms of congestion were thus relieved, but the palsy continued to make progress slowly. In the second case, the hereditary character of the disease was also shown, and more and more frequent and violent attacks of cerebral congestion were induced by the suppression of an epistaxis. The disease in this case was only commencing. The third case was that of a Pole, forty-three years of age, much addicted to drinking and to venery. The disease was also hereditary in this case. Eight years prior to coming under notice, he had had an attack of apoplexy, followed by hemiplegia, respecting which M. Lunier observes, it is not uncommon to see progressive general palsy follow hemiplegia, the result of one or more attacks of cerebral congestion. In such cases the hemiplegia progressively disappears, but at the end of a certain time, if the patient be carefully examined, it will be found that the palsy is not so clearly defined; there are irregularities, and one of the limbs of the other side presents slight symptoms of palsy, or perhaps the sensibility is greater in the arm of one side and the leg of the other, or the patient sees better with one eye, and hears better with the opposite ear. These singular anomalies form the transition, so to say, of the hemiplegia into general palsy. When the paralysis begins to become general, the hemiplegia becomes stationary: this is one of the signs which should make us dread the occurrence of general palsy in the hemiplegiac; when the general palsy is fully established, the hemiplegia, instead of progressing towards a cure, increases in intensity, and follows the progress of the general disease. In those persons in whom general palsy has thus succeeded hemiplegia, there is always, even in the third stage of the disease, a predominance of the palsy in the side primitively affected. The patient in the fourth instance was a young man, eighteen years of age, in whom the disease supervened in consequence of a fall from a height of several feet, on the left side of the head, by which concussion of the brain was caused. In this case, M. Lunier considers the immediate cause of the general palsy to have been acute hydrocephalus become chronic. M. Requin has published a case of progressive general palsy following an injury to the head, which ultimately terminated fatally. The autopsy disclosed evidences of chronic meningitis. The fifth case is one previously published by M. Brierre de Boismont; and the sixth is that recorded by M. Delaye, already alluded to.

The second series of cases, in which are recorded those in which the progressive general palsy was not preceded by insanity, but was afterwards accompanied by dementia, are four in number. The complication or occurrence of dementia, M. Lunier regards as an almost constant symptom of the disease, when it has reached a certain stage, and he believes it to depend on the same organic cause as the lesions of motion and sensation. The first case is that of a woman, fifty years of age, in whom again the disease was hereditary. The disease advanced to the second stage, was accompanied by symptoms of dementia, and then became stationary. The second case is an instance of a predisposition to cerebral congestion, with intermittent and alternative palsy of the arms consequent on hard labour and general progressive palsy. The patient was affected with suicidal lypemania. The man was thirty-five years old. The third is an instance of general palsy in the commencement of the second stage. The disease was hereditary. The abuse of mercury was considered to have had something to do in causing the disease; but this M. Lunier will not admit, and seems more inclined to refer it to the abuse of tobacco. He says that great smokers are peculiarly predisposed to cerebral congestion, and consequently to general paralysis. The question he thinks worthy of further examination.

In the second stage of general paralysis, and sometimes even in the third, the vegetative functions are generally well performed. The appetite is good, often even rather voracious, and the patients present some *embonpoint.* Emaciation, marasmus, and gangrenous spots occur later in the disease. M. Baillarger was the first to point out the loss of the generative power in the commencement of the disease. M. Lunier observes that he has noticed it in several cases. It is a premonitory symptom of some importance, but in some cases there occurs a kind of venereal orgasm, which may be present, although rarely, even in the third stage of the disease.

The loss of the memory is undoubtedly the beginning of dementia. It is often the only sign of intellectual debility that can be discovered during the first and even the second stage of general palsy.

The fourth and last case in this series is one in which the disease was hereditary, and was attended by two attacks of ambitious delirium. It was ultimately complicated with dementia.

The third series in which the general paralysis was, or seemed to be, preceded by mania or monomania, transformed or not afterwards into dementia, consists of one case only. The patient, a man fifty years of age, had a fall from a height of eight or ten feet on the back part of his head, a few days after which he was admitted into the Bicêtre with symptoms of mania. M. Lunier is of opinion that at this time the patient was labouring under unrecognised general paralysis. At the date of the report, ten years after the accident, the man did not present any signs of dementia.

Hallucinations in Early Infancy.—Dr. Thore, jun., in a communication published in the *Annales Medico-Psychologiques*, details the case of an infant, fourteen months and a half old, who had been poisoned by the seeds of the datura stramonium. In this case, hallucinations of the organs of sight were well marked, as evidenced by the motions of the child. She appeared to be incessantly seeking for imaginary objects in front of her: she stretched out her hands to get at them, and clung to the sides of her cradle to reach them the better. While these hallucinations were the most marked, actual vision appeared to be abolished, thus affording an additional proof that the integrity of an organ, which becomes hallucinate, is not necessary in order that the false sensation may occur.

M. Thore observes, that those medical men who have especially devoted themselves to the consideration of insanity, have not hitherto studied much the hallucinations which occur during the early periods of life. Examples after seven years of age are met with in their writings, but they are few in number. Nevertheless, they are frequently noticed even before the age just mentioned, and are developed under the influence of greatly differing causes. It is not uncommon to notice them in the course of acute diseases. M. Thore has frequently met with hallucinations of the sight and hearing in children of from four to five years of age. He adds, that they are of occasional occurrence in children, even while in perfect health. They then appear to form part of a previous dream.

Prolonged Bathing and Continuous Irrigation in the Treatment of the Acute Forms of Insanity.—M. Brierre de Boismont advances the following propositions as among the results of this practice:—

1st. The greater slowness of the circulation and respiration—the introduction of a large quantity of water into the economy—the general and gradual refrigeration, show that these baths have an essentially calming and sedative action.

2nd. The period of convalescence ought to be carefully watched, because relapses are not rare, when the patients are too suddenly exposed to the influence of the causes which have occasioned the disease.

3rd. When acute mania is complicated with acute delirium, and the patient refuses drinks, the treatment is inefficacious.

4th. Prolonged bathing and continuous irrigations appear to M. Brierre de Boismont to be useful in hysterical affections, and many other nervous diseases with excitement.

5th. Prolonged bathing is without its disadvantages; the fatigue it causes soon disappears; it does not deprive the system of any important principle, nor leave behind it that utter debility so often caused by free bleedings, and which has more than once terminated in dementia.

M. Brierre de Boismont's treatment by the plan above indicated appears to have been very successful.—*Gazette Médicale.*

Endemic Cerebro-Spinal Meningitis.—M. Corbin, of Orleans, has published in the *Gazette Médicale* an account of an endemic cerebro-spinal meningitis, which affected the garrison of that city in 1847-48. The following are his conclusions:—

1st. In the disease called cerebro-spinal meningitis, which has raged epidemically for ten years, especially in certain garrisons, the inflammation sometimes extends to the nervous centres, principally to the spinal marrow, which becomes softened. It is a cerebro-spinitis, or more correctly, a meningo-myelitis, a meningo-encephalitis.

2nd. The disease prevails principally during the winter, and appears to depend chiefly on the overcrowding (*encombrement*), the alteration of the atmosphere, perhaps from a too high artificial temperature: whence there results, as a means of prevention, the necessity of inverse hygienic conditions.

3rd. The treatment ought to be antiphlogistic and revulsive.

4th. Special circumstances, which have been called the epidemic character, may require a special medication. Opium was very useful in the treatment of the disease during the latter part of the time that the epidemic prevailed in Avignon.

Experiments on the Respiratory and Arterial Motions of the Brain.—M. Flourens had hitherto in his experiments only recognised the respiratory motion of the brain; he denied the arterial. Additional researches have since convinced him that, as Haller had already indicated, there exist, in fact, two motions of the brain:—

1st. The respiratory, which all anatomists attribute to the alternating flux and reflux of the venous blood. But while Haller and Lamure profess that these flux and reflux are effected by the jugular veins, M. Flourens believes that the principal source of the venous blood, which by its reflux causes the swelling of the brain, is to be found in the two great vertebral venous sinuses.

2nd. And the arterial, which, according to M. Flourens' experiments, depends on the influx of the arterial blood, which is sent to the brain on each contraction of the left ventricle of the heart.—*Gazette Médicale.*

Hysteria.—At a meeting of the *Société Medico-Pratique* of Paris, M. Michea detailed a case of hysteria, as follows:—

The patient, twenty-five years of age, of a robust constitution, and apparently sanguine temperament, had a first attack of hysteria during her first pregnancy. When summoned to see her, the attacks having become very frequent, M. Michea ascertained that there existed insensibility of the skin and mucous membranes, without palsy of motion. The urine of the patient clear, slightly acid, without sediment, presented only a rose colour when treated with heat and nitric acid. Under the microscope it presented a diminution in the quantity of urea and uric acid. A thousand parts of the blood, when analysed, showed—water, 804; globules, 74; fibrine, 2; solid matter, 118. There was, therefore, a diminution of the quantity of red globules.

Treatment of Hysteria and Chlorosis.—M. Delfrassé, of Cahors, writes to the National Academy of Medicine, that he has derived great benefit from the use of the acetate, or hydrochlorate of morphia, passed into the cavity of the uterus, in cases of hysteria, chlorosis, and other nervous affections.—*Bulletin de l'Académie Nationale de Médecine.*

Chronic Sciatica.—M. Robert has published in the *Annales de Thérapeutique Médicale et Chirurgicale*, the case of a countrywoman, fifty years of age, who had suffered for fifteen months intense pains in the right lower extremity, extending from the sciatic notch to the foot. The pains were greatly aggravated at night, so that she could not rest in bed nor obtain sleep. Various plans of treatment had been tried unavailingly. M. Robert, after chloroform had been administered, applied an iron at a white heat on the outside of the dorsum of the foot, in the direction of a line drawn from the external malleolus to the third and fourth toes. When the patient awoke, she declared the pain had entirely ceased, nor had it returned at the date of the report, a fortnight after the operation.

Sonnambulism.—Professor Vannoni describes in *Il Progresso* a singular case of sonnambulism. He was summoned to see a young married lady, and ascertained that she was pregnant. This declaration evidently caused her extreme pain, the explanation of which was not made to him until a few days afterwards, when it appeared that the husband, to whom she was greatly attached, declared he could not be the father of her child, as he had not had intercourse with her for five months. From some remarks that fell from the lady, it was discovered that the husband had become a sonnambulist, and that the attack came on about one o'clock each morning, when he left his own room and visited his wife unconsciously. The sonnambulism appears to have arisen in consequence of alarm caused by an accident to his mother.

Aconitum Napellus.—M. Teissier, of Lyons, has conducted a series of experiments on the aconitum napellus, with the view of studying its stupefying and antiphlogistic actions. The stupefying action is undoubted; it differs from that of morphia, the influence of which is perceptible in more or less relieving all kinds of pain; aconite, on the contrary, has power only over special pains. This specialty of action of the aconite is one of its principal characters, and it results from the fact that the stupefying property of this medicine is only secondary: its principal, and in some sort specific, action is exerted on the skin; it consists in eliminating the noxious elements from the vessels of that membrane, and in re-establishing its functions, when they have been disturbed either by the repercussion of the perspiration or by the presence of any virus. Thus aconite is adapted for the treatment of diseases caused by cold, the consequences of catarrhs, and also of the diseases in which a morbid principle is retained in the cutaneous tissue, such as the exanthematous fevers. The painful diseases in which M. Teissier has obtained benefit from the sedative action of aconite, are those depending on a catarrhal or rheumatic cause. The antiphlogistic action of the plant is quite secondary and subordinate to its action on the skin.—*Révue Médicale.*

Chloroform in Neuralgia.—M. Barrier, of Lyons, and Messrs. Rollet and Jeannel, of Bordeaux, have employed chloroform successfully in the treatment of neuralgia. The first-named administered it by inhalation; the others employed it topically, a flannel moistened with forty drops of chloroform being applied to the affected part. The local action of chloroform has been compared to that produced by a sinapism. M. Amesille, at a meeting of the Academy of Medicine, stated that he also had had recourse to the topical application of chloroform, with benefit in diverse cases of neuralgia,—in a case of suffocating, precordial pain, and in two cases of very severe nervous colic.—*Revue Médicale.*

Treatment of Epilepsy.—M. Cheneau, in a communication read before the Academy of Sciences at Paris, in summing up his remarks on the treatment of epilepsy, observes that the cure of that disease, when it takes place, is not exclusively at the early period of life, but may be effected at a rather advanced age : that the difference of age does not modify in a notable manner the chances of success, nor the difficulty of the treatment; that the complications of idiocy, and more or less extensive palsy, are not insurmountable obstacles to the cure; that the treatment may be of short duration, but that it is impossible to ascertain this beforehand ; that digitalis is worth the attention of practitioners in the treatment of this disease; that the opinion emitted by physicians that several fits cannot occur in the same day is not sufficiently supported; that it is an error to look upon an extreme paleness, succeeding towards the close of the fits the redness which had previously existed, as a characteristic symptom of epilepsy ; and that man is subject to a kind of epilepsy, called *tournis*, which is not always dependent on a lesion of the brain, or of the peduncles of the cerebellum, and may be cured.—*Gazette Médicale.*

Hydrophobia.—Dr. Jackson, of Philadelphia, has published several cases of hydrophobia in the *American Journal of Medical Sciences*, in one of which chloroform was administered with relief to the spasms, but the case terminated fatally. In another case, the cicatrix of the bite was excised, the wound cauterized, and free suppuration induced. Chloroform was also used whenever the spasms threatened. The patient recovered. Dr. Jackson appears to be doubtful whether or not the case is to be considered one of hydrophobia. Dr. Curtis, of Lowell, has published in the same journal another case of hydrophobia, in which chloroform entirely failed.

Epilepsy.—Dr. R. W. Evans, of Richmond, C. W., relates ("British American Journal of Medical and Physical Science,") a case of epilepsy successfully treated by an infusion of the *scutellaria geniculata*, made according to the following formula: ℞ Scutell. genic., ʒij ; aq. bullientis, f ℥viij ; ft: inf. The mode of administration is to begin with two tablespoonsful every eight hours, increasing the dose, after the termination of a week, to double that quantity, with an occasional aperient.

The subject of Dr. Evans' case was a female, 26 years of age, who had suffered from the disease for eight years, the attacks coming on every six or seven days. She had taken for months nitrate of silver, iron, zinc, strychnia, digitalis, ammoniuret of copper, valerian, musk, &c., without any benefit. Dr. Evans directed the scutellaria according to the above formula ; its use was continued for six weeks, when a profuse salivation took place, with slight constriction of the fauces. The medicine was discontinued, a Seidlitz powder directed, and in a few days the ptyalism ceased At the date of the report, the patient had been taking the medicine daily for four months, during which time she had not had a single attack. She enjoys excellent health, and her memory seems to improve daily.

Dr. Evans states that he has under his care two other cases, which seem to manifest the superiority of this medicine. They are in a manner almost recovered, with the exception of a violent palpitation of the heart at the expected period of attack, which passes off without any bad result by the timely administration of a few doses of tincture of digitalis, and by keeping the patient free from mental irritability, which is a frequent cause of epileptic palpitation.

The Essential Pathological Conditions of the Brain in Insanity.—Dr. Palmer, of Bow, in a communication published in the "Lancet," says, no evidence is wanting to prove that the vesicular neurine of the convolutions is the centre of the intellectual operations, and that, if it be not also the emotional centre, it is, at least, most intimately connected with it. Here, by means of some molecular changes, our sensations first become perceptions, impressions are fixed, and trains of thought are worked out in the various operations of imagination, abstraction, comparison, &c. This view of the seat of the intellect is held by nearly all great anatomists and physiologists, and may be regarded as a settled point; we have thus a strong light to guide us in the investigation of the pathology of mental diseases, and are led unhesitatingly and necessarily to refer all intellectual unsoundness, of whatever form or grade, to some deficiency of, or morbid molecular change in, the vesicular neurine of the convolutions. The remote causes of insanity may be either physical or moral, eccentric or centric, originating in other organs of the body or operating immediately on the intellectual centre; but to whatever sources they may be traced, some disturbance of the healthy molecular changes on which the nutrition and functions of the cortical grey matter depend, must be presupposed before insanity can be admitted. The appearances which have come under Dr. Palmer's observation, in the post mortem examination of the brains of eighty-two lunatics, are quite in harmony, he says, with these views, not one case having been met with in which there was not distinct lesion of this portion of the grey matter, associated in most cases with other morbid conditions of the brain or other viscera, which had their corresponding symptoms during life, but always bearing a direct ratio to the extent of mental aberration. Cases of insanity, he adds, may, and no doubt do occur, in which no traces of cerebral disease are discoverable after death, but this cannot be received as evidence that such disease did not exist during life, and was operating as the immediate cause of the mental aberration.

Various degrees of defective development, anemia, atrophy, &c., of the cortical matter, are commonly met with, but the most striking and apparently specific lesion in insanity is asthenic inflammatory degeneration of this substance, to which may probably be referred all the alterations in its colour and consistence, noticed from time to time by various writers. It proceeds from without in an inward direction, commencing immediately beneath the pia mater, and advancing until the entire thickness of the cortical substance is involved. On different parts of the hemispheres it may be observed in different stages of its progress, but always more definitely marked about the apex of the posterior lobes, in the posterior part of the convolution, described by Solly as the great convolution of the second order. It is also often observed in the middle lobes, and occasionally, but less distinctly, in the frontal lobes. On minute examination, a well-defined line is perceived to intervene between the outer degenerated portion, which has already succumbed to the inflammatory action, and is of a pale, ash-grey colour, without any appearance of vascularity; and the inner, which is of a full, brownish, pink hue, highly injected, and distinctly undergoing the inflammatory process. Dr. Palmer has not examined these structures under the microscope, but he says he has no doubt that complete disorganization of the vesicular neurine would be found in the ash-grey layer.

Insanity—i. e., disease of the convolutional grey matter, has not of itself a fatal tendency, but death usually results from some previously existing, associated, or induced disease of other portions of the cerebral mass, or of other organs. The nutrition of the cortical matter is often primarily disturbed by the influence of morbid blood circulating through it, and as often by the influence of distant visceral disease conveyed to it through the medium of the sympathetic grey matter of the cord, olivary columns, sensory tract, and radiating fibres of the hemispheres. The reverse also frequently takes place—viz., the cause of the intellectual disturbance being purely moral, the quantity and quality of the secretions, and the process of nutrition generally, may be morbidly altered, so that the blood becomes changed in its properties, and vital organs impaired in their power of resisting morbid impressions. Both these conditions are of common occurrence, and constitute distinct varieties of insanity in regard to symptoms, prognosis, and treatment, although the cortical matter probably undergoes the same change in each of them.

Watchfulness, and a rapid flow of ideas, with emotions not readily under control, are the more prominent mental phenomena of the first stsge—that of undue irritability of the grey matter. In the second, that of erethism, (also the stage of invasion,) a rapid succession of illusions and false perceptions, and their quick obliteration, are the more salient features. These, however, are always in proportion to the amount of cortical matter implicated. As structural lesions take place, the illusions and perversities of character become less transitory, and are more or less numerous in proportion to the superficial extent of disorganization, and more or less permanent and complete in proportion to the depth to which the cortical matter is diseased. This constitutes chronic insanity, which, when the inflammatory action ceases, (as is often the case,) may be termed fixed chronic insanity, in contradistinction to the still extending disease, which may be designated progressive chronic insanity. These forms are very distinct in symptoms, as well as in the treatment required. In the former, there are certain fixed delusions and oddities of character without the watchfulness, excitability, and hurry of ideas characteristic of erethism; in the latter, these fixed delusions are still prominent, but the indications of erethism are superadded. In the former, the general health may remain good for years; in the latter, the organic nervous system may soon become deranged, and the patient fall a victim to some abdominal or thoracic disease. At length, when the vesicular neurine of the convolutions is both extensively and deeply disorganized, the various grades of dementia, ending in a total obliteration of the mental faculties, supervene.

Another form of dementia of an acute character is sometimes observed to follow immediately on the erethismic stage, without passing through the grades of progressive chronic insanity. It is not unfavourable as regards prognosis, and is probably due to the capillaries relieving themselves from congestion by inter-vesicular effusion, (edema of the grey matter,) and thus causing suspension of the intellectual functions by mechanical compression of the vesicles. Diminished nutrition, and vital stimulus of the grey matter from imperfectly filled capillaries produces simple delirium, (present in the course of many exhausting diseases,) but if long-continued, and especially if the blood be in an impoverished state, it also furnishes one of the pathological conditions of senile insanity.

Hemicrania.—Dr. Turenne states that hemicrania is a pain in the head resulting from the compression of the trifacial nerve, more particularly of its ophthalmic branch, caused by the accumulation of blood in the sinuses at the base of the cranium, and especially in the cavernous sinuses.

Origin of the Vis Nervosa.—Mr. Strachan, in a letter published in the *Lancet* on the origin of the vis nervosa, observes that, by every chemical action either positive or negative electricity is produced. It is clear, therefore, he says, that the various and extensive chemical actions constantly going on in the animal system—the change from venous to arterial blood in the lungs—the change from arterial to venous blood in the capillaries—the various secretions—the digestion of food—the nourishment and decay of every organ, are sources whence an immense amount of electricity must be evolved. And for the hypothesis that this is the source of the nervous fluid, it is only necessary to suppose that the afferent nerves receive the electricity set free, and convey it to the brain, whence it is constantly returned by the efferent nerves, and thus an electric circle is produced, which again modifies all the chemical changes going on, and is capable of producing all the effects usually ascribed to the nervous fluid. According to this view, the brain and spinal marrow, instead of generating, serve the purpose of accumulating, distributing, and directing the nervous force. They hold a similar relation to the nervous fluid that the heart does to the blood. Or they may be likened to the coiled wire of the galvanic machine.

Chloroform in Delirium Tremens.—Mr. Hyde, of Maryborough, records in the *Lancet* a case of delirium tremens, which was apparently cured by the production of anesthesia. About a drachm and a half of chloroform were used. The anesthetic condition continued about eight minutes, soon after recovering from which, the patient fell into a quiet, deep sleep, in which he remained for nearly fourteen hours, when he awoke with clear and sound intellectual faculties. The case afterwards did well. Opiates were given before the chloroform was employed.

Chloroform in Quotidian Hemicrania.—Mr. Broxholme, of Islington, records in the *Lancet* a case of severe quotidian hemicrania, followed by an attack in some degree resembling asthma, in which he successfully had recourse to the anesthetic action of chloroform. Aperients and subsequently quinine were also used.

Puerperal Convulsions.—At a meeting of the Westminster Medical Society, Dr. Cormack detailed the history of three cases of puerperal convulsions which had occurred in his practice. The main object of his paper was to point out the connexion between renal congestion and puerperal convulsions, which exists in a very great proportion of cases. He considered puerperal convulsions to be, though not always, yet generally, the toxicological results of non-elimination of the excretion of the urine; and that in by far the greater number of cases this non-elimination depends on the renal congestion, caused by the pressure of the gravid uterus. Edema and albuminuria are frequently concomicants or precursors of convulsions, as shown by Dr. Lever and Messrs. Devilliers and Regnault. The gravid uterus, or any tumour pressing on the renal veins, must cause congestion of the kidneys, and consequent toxemia; this is the more injurious to the pregnant woman, as her blood requires an extra degree of depuration, both from the excrementitious matter from the fœtus, and also from the elements of milk. Retention of these should, Dr. Cormack thought, be considered as the cause, not only of convulsions, but also of various other distressing symptoms occurring during pregnancy. Uterine epilepsy probably often arises from toxemia, and the suppression of the lochia may induce *post partum* puerperal convulsions. When convulsions recur after delivery, we must suspect structural renal disease. The explanation of delivery generally arresting convulsions is not so much that uterine irritation is lessened, as that the hyperemic state of the kidneys is relieved. The more common subjects of puerperal convulsions are strong, healthy young women, pregnant for the first time. In them the abdominal walls are most unyielding, and unable to relax under the pressure of the gravid womb. Cases of puerperal convulsions in subsequent pregnancies may be either toxemic or non-toxemic; the toxemic cases may be classed under the following heads:—1st. The persons who have never gone the full time; 2nd. persons of extreme muscular development; 3rd. persons suffering from structural disease or obstruction of the kidneys; 4th. excessive volume of uterine contents, including twin cases, &c.—*Lancet.*

Lunatic Asyla in the United States.—Mr. Thornton, of West Ham, in a letter published in the *Lancet*, states that the proportion of cures of insanity is greater in America than in England. With few exceptions, the American physicians regard all chronic cases as susceptible of cure, or of much improvement, and that large class, usually called incurable, are treated with a perseverance which is often rewarded with a successful issue. Dr. Kirkbride believes that of all who are attacked with insanity, and subjected during its early stages to judicious treatment, faithfully persevered in, at least eighty per cent. will recover. Many cases, Mr. Thornton observes, believed to be incurable, are placed in confinement for safety only; and thus condemned to the exclusive society of incurables, whereby any chance of returning reason is frustrated. Every intelligent person knows the effect of association upon his own mind; he feels in himself and sees in others that moral and refined society have an elevating influence, while the companionship of the profligate, ignorant, and deluded bring down the standard of the mind, and destroy its natural balance. Hence moral means, which are to aid in the restoration of curables, are often useful in keeping up the mental powers of those who may be incurable.

The chief excellence of the treatment in the asylums of the United States consists in carefully classifying the patients, and in arranging the inmates of the separate wards, so as to exclude those cases, the subjects of which may in any way interfere with the progress of the majority; and those who are practically conversant with the treatment of the insane will admit that this class separation is as imperative for insuring the mental restoration of the insane, as individual seclusion is necessary for improving the moral condition of the criminal.

